# Real Time Estimator to Perform Targeted Biopsies With a Free-Wrist Robot Despite Large Deformations of the Insertion Orifice

**DOI:** 10.3389/frobt.2021.780505

**Published:** 2021-11-15

**Authors:** Rémi Chalard, Afshin Fazel, Marie-Aude Vitrani

**Affiliations:** ^1^ AMPERE Laboratory, CNRS UMR 5005, INSA de Lyon, Lyon, France; ^2^ Departement de Gynécologie Obstétrique, Hopital Lariboisière, Université Paris Diderot et de l’APHP, Paris, France; ^3^ Institut des Systèmes Intelligents et de Robotique (ISIR), CNRS UMR 7222, INSERM U1150, Sorbonne Université, Paris, France

**Keywords:** uterine biopsy, probe holder, reaching task, online identification, robotic

## Abstract

In the context of keyhole surgery, and more particularly of uterine biopsy, the fine automatic movements of a surgical instrument held by a robot with 3 active DOF’s require an exact knowledge of the point of rotation of the instrument. However, this center of rotation is not fixed and moves during an examination. This paper deals with a new method of detecting and updating the interaction matrix linking the movements of the robot with the surgical instrument. This is based on the method of updating the Jacobian matrix which is named the “Broyden method”. It is able to take into account body tissue deformations in real time in order to improve the pointing task for automatic movements of a surgical instrument in an unknown environment.

## 1 Introduction

During minimally invasive surgery (MIS), instruments and imaging devices are inserted into a patient through small orifices. The orifice can be artificial, e.g., during laparoscopy where cannulas are placed through the abdominal wall. It can also be natural, e.g., during a vaginal manipulation where a manipulator (and/or an ultrasound probe) is inserted through the patient’s vagina.

When an instrument is inserted through an orifice, forces appear at the insertion area and induced mechanical constraints. In order to guarantee the patient’s safety during a robotic keyhole surgery, these forces should be minimized and the most commonly solution is a kinematic solution. More precisely, inserting an instrument through an orifice is equivalent to rigidly constrains the movements of the instrument along 4 degrees of freedom (DOFs): one translation along the axis of the penetration and three rotations around a given point *R*. This kinematic constraint come from the assumption that the body stiffness in an orifice is maximal at an anatomical point *A* located a few millimeters under the body surface. Therefore, to minimize the forces at the insertion area it is necessary to achieve *R* = *A*.

Numerous solutions are implemented in the literature to cope with the kinematic constraint due to the insertion of the instrument through a cavity such as in laparoscopy or during prostate biopsies, etc. But most of them assume that the insertion point plays the role of a 2-DoF kinematic constraint. For example, it is the case for the 4-DOF robot exhibiting a remote center of motion (RCM) (Guthart and Salisbury, 2000; Wei et al., 2005) which needs a pre-operation placement prior to the instrument manipulation. Another solution is to use a fully actuated robot such as in (Schneider et al., 2004; Konietschke et al., 2009) where the kinematic constraint is solved by the robot control. This solution is currently used because it does not require a specific placement in the operating room but a registration of the insertion point is still necessary prior to the operation (Boctor et al., 2004; Dombre et al., 2004; Pham et al., 2015). The main limitation of all these approaches is that they use a model which does not always correspond to the reality. Indeed, in many cases, due to the deformation of the insertion area, minimizing the interaction forces at the entry of the instrument is not equivalent to perfectly pivoting around a fixed point. In different works, (Chalard et al., 2018; Smet et al., 2019), it has been shown that during different kinds of MIS it is not possible to consider the insertion area as a fixed point *A* around which the instrument rotates. Therefore, using solution as an installation calibration, registration, or control appears as not appropriated to deal with the minimization of the forces at the insertion area of the instrument. To cope with this assumption, the free-wrist robots (a spherical wrist without actuators, (Sackier and Wang, 1994; Munoz et al., 2000; Ortmaier and Hirzinger, 2000; Low and Phee, 2004)) are of great interest. With these devices, as the robot lets the instrument freely orient around the wrist center W, the insertion point constraint is automatically respected when the instrument tip is inserted into the patient. Moreover, the wrench applied to the patient at the insertion point is naturally minimized. However, a main drawback occurs when a precise location is to be reached by the instrument tip *T*. In such a situation, the robot positions its wrist center W in order to manipulate the tool from outside the patient. Obviously, the position of the tool tip T inside the patient results not only from the position of point W but also from the location of the so-called insertion point. In practice, one can rarely rely on the definition of a fixed insertion point, as backlash or deformation of the tissues surrounding the insertion area occur. This is particularly true for the uterine manipulation (Smet et al., 2019) which is one of the application of this paper.

The paper is as follows: *Materials and Methods* describes firstly the proposed procedure to biopsy deeply the uterus. The second part of the *Materials and Methods* is focused on the overall system to assist the gesture. Then, based on the anatomical description and other work, (Smet et al., 2019), robot specifications are defined and a robot probe-holder is chosen. This robot is an anthropomorphic arm with 3 actuated joint and a free wrist. Because of the free wrist, precise positioning requires the estimations of the kinematic constraint due to the interaction between the probe and the tissues. Two online model estimation based on the Adaptable Lever Arm Model (ALAM) and the Broyden method are described and tested in *Precise Positioning* and *Simulation Results*. It is implemented on a robotic control law in order to accurately position the probe tip. Finally, *Results* highlights different results validating our approach.

## 2 Materials and Methods

### 2.1 Proposed System

#### 2.1.1 New Uterine Biopsies Procedure

**FIGURE 1:**
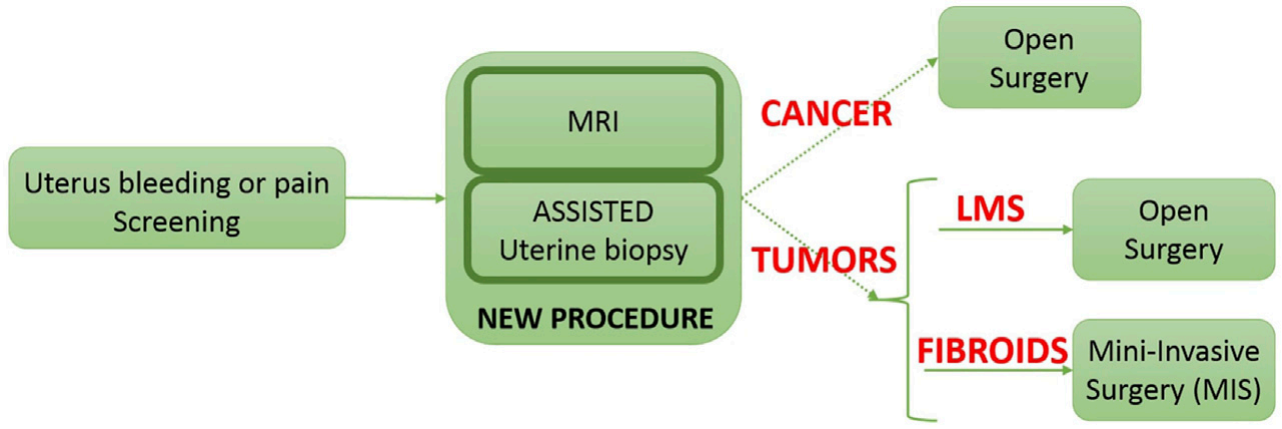
The new procedure which is able to identify the fibroids classification.

**FIGURE 2:**
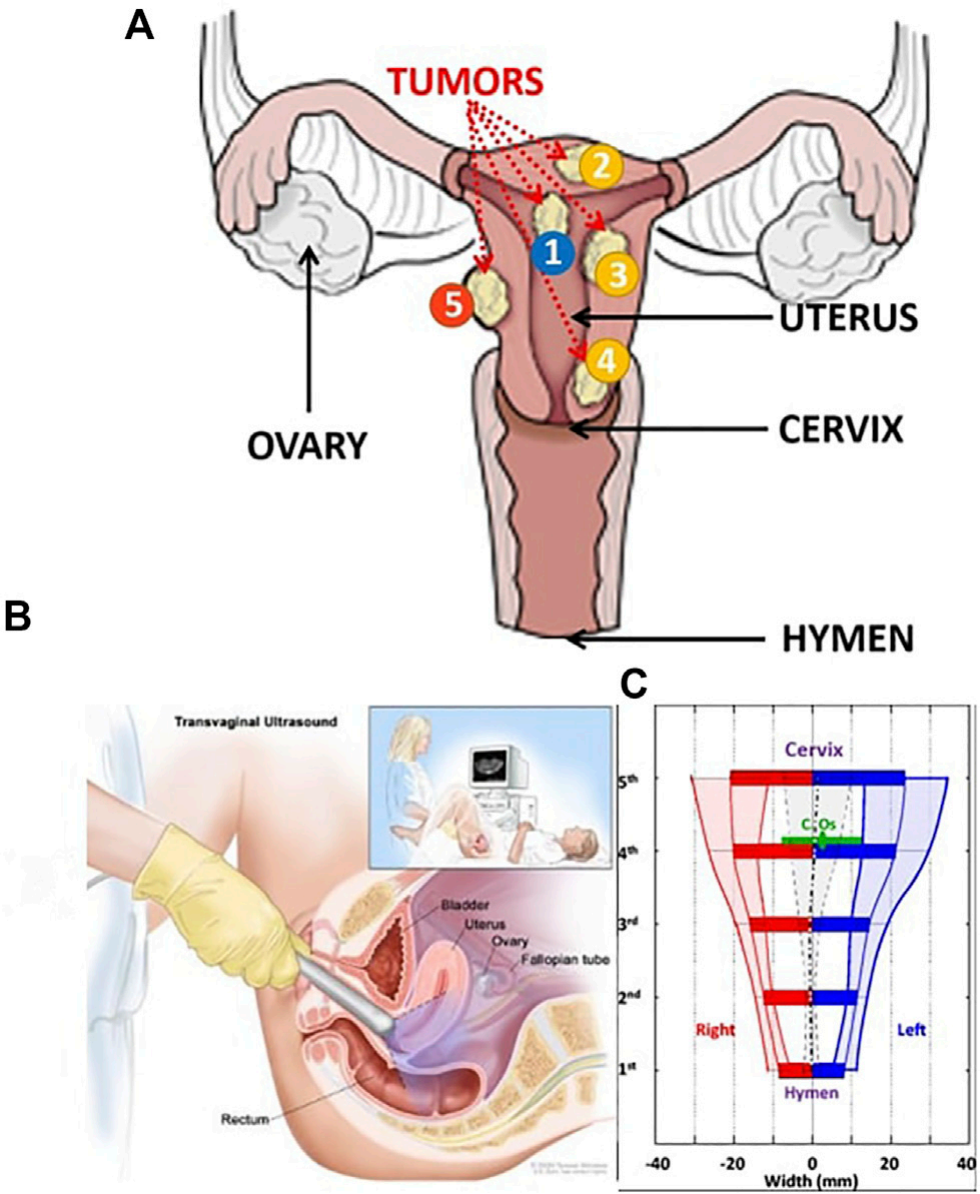
**(A)** Uterus description with all types of tumors (Ochsner, 2021),modified. In blue (label 1) it is the submucosal tumors, in yellow (label 2,3,4) the intramural tumors and in orange (label 5) the subserosal tumors. **(B)** Clinical routine for transvaginal echography and **(C)** Vaginal measured description (Luo et al., 2016) modified.

There are a number of tools used by clinicians to diagnose women with tumors. It included physical exam, serum biomarkers, sampling/cytology, ultrasound (US), hysteroscopy, hysterosalpingography, magnetic resonance imaging (MRI) and computed tomography imaging. However, the only gold standard to distinguish a malignancy from a benign condition is a biopsy. Until now, only endometrial sampling performed. Endometrial biopsies may not provide the correct diagnosis unless the lesion has reached the surface of the endometrial cavity (Van der Bosch et al., 2012). These uterine biopsies are performed thanks to a hysteroscope inserted through the vagina into the uterus (Tamura et al., 2015). This procedure allows to sample only the tumors visible in the uterus cavity (submucosal tumor). It cannot be used to sample deeply in the uterus (subseral and/or intramural tumors), see Figure 2. In case of uterine fibroids, several studies (Van der Bosch et al., 2012), (Bansal et al., 2008), demonstrate the importance of exploring deeply the uterus in order to specify whether an observed tumor is benign or malignant. A targeted uterine biopsy system appears as essential (Kawamura et al., 2002), (Tamura et al., 2015) to reach prior to laparoscopic surgery of any uterine mass (see Figure 1). However, there is no routine tool allowing reliable deep sampling in the uterus. It requires the development of innovative functions exploiting state of the art in imaging and robotics to enable a secure, reproducible, and accurate sampling.

A study in progress (Tamura et al., 2015) on 63 patients concludes that ultrasound-guided needle biopsy may be a reliable preoperative diagnostic procedure for uterine tumors with suspected malignancy by MRI. The proposed approach, detailed in (Fazel et al., 2016), is based on trans-vaginal ultrasound needle biopsy. This procedure is similar to the procedure to sample the prostate under transrectal ultrasound images (Vitrani et al., 2016). During the proposed intervention, the patient lies on gynecological position. A trans-vaginal ultrasound probe and a needle guide attached to it are inserted in the patient’s vagina. Then, the clinician moves the probe toward a first desired biopsy site. When they think that the probe is well positioned, the clinician can proceed to the biopsy by inserting the needle through the needle-guide. They repeat the above procedure until all the biopsies have been done.

**FIGURE 3:**
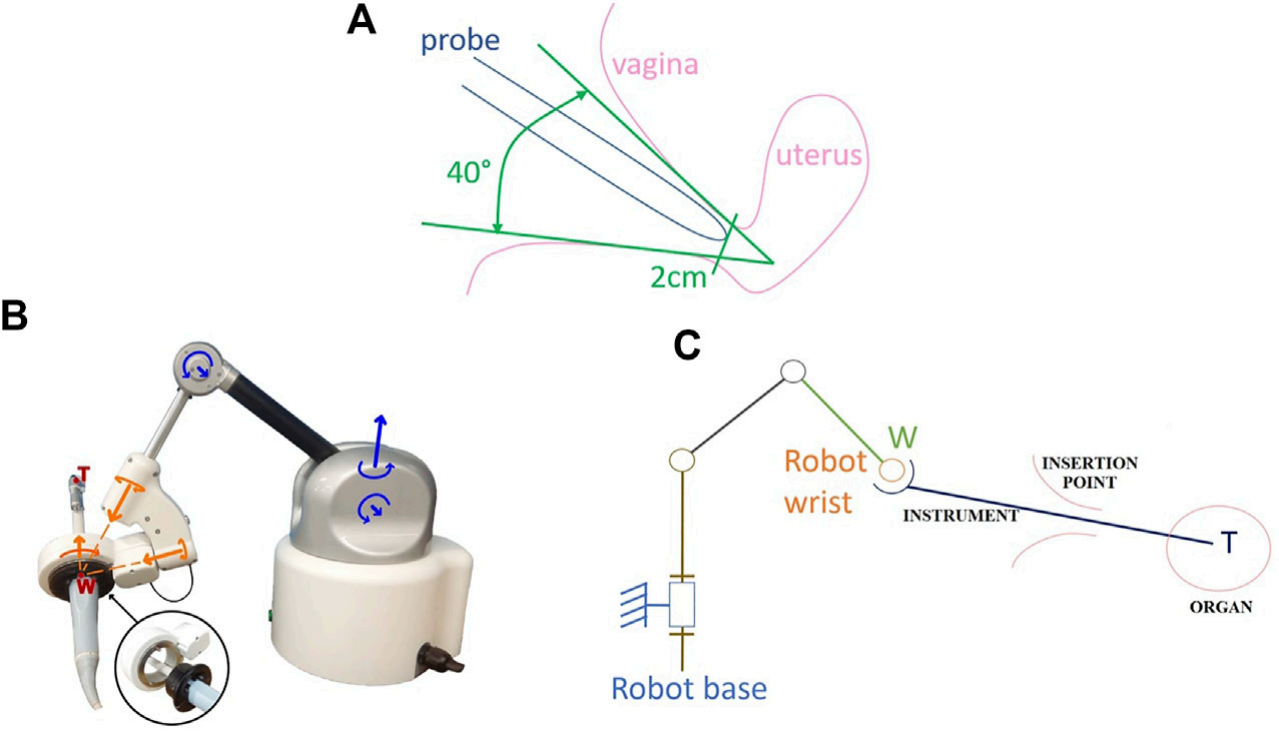
**(A)** Required workspace to manipulate the probe within the vagina **(B)** Apollo robot and **(C)** Kinematics scheme (Poquet et al., 2013).

To reach each position, the probe is inserted through the vagina and its tip is in contact with the cervix (base of the uterus) which anatomy is described in (Bouton et al., 1990) (see Figure 2). According to surgeons, the probe tip has a small mobility within a 1 cm radius circle limited by the cervix and the vaginal wall, (Smet et al., 2019). Furthermore, the overall probe has to be moved in many orientations limited by the vagina wall. The overall workspace of the probe can be modelled by a truncated cone with 40° top angle, Figure 3.

#### 2.1.2 Robotic Specification

To our knowledge, only two robots are proposed to manipulate a probe within a vagina, (Akrivos and Barton-Smith, 2013) and (Yip et al., 2017). Both of them are used for trans-vaginal uterine manipulation which does not require high precision. However, for uterine biopsy, the surgeon has to precisely position the probe (and the needle) while maintaining minimal effort on the cervix and the vagina (insertion zone). The robot control law must take into account the displacement and the deformation of the vagina. The mechanical-based RCM strategies can’t be relevant in our work. One can refer to two particularly interesting studies: the system presented in (Bonneau et al., 2004) which does not use any force sensor and the work of (Abolmaesumi et al., 2002). These systems focus on an ultrasound probe holder controlled by machine-vision to center a section of the carotid in the image: 3 degrees of freedom are controlled by machine-vision while the operator can control the other 3. An other work on robot-assisted ultrasound-guided biopsies have shown similar degree of precision in robot assisted transrectal prostate biopsy described in (Poquet et al., 2015). This is why we propose to use a comanipulated robot to assists the clinician’s gesture.

#### 2.1.3 Robot Apollo

Apollo (Figure 3) fits in the category of the free-wrist comanipulators, although it differs from the existing systems by the functions it provides (Poquet et al., 2013). Instead of separating between robotic autonomous probe placement and human needle placement, it lets the clinician position the probe. This choice is motivated by the difficulty of planning a trajectory for the probe positioning when accounting for uterine displacement, eventual movements from the patient, anatomical constraints, etc.

It exhibits 6-DoFs to be compatible with all the required probe movements (*Robotic Specification*) while avoiding to constrain its placement with respect to the patient. While the robot base is placed close to the entry point, on the examination table, it allows the probe to cover the required workspace. This workspace was determined based on the clinical literature, (Bouton et al., 1990), (Tan et al., 2006), (Luo et al., 2016). Apollo is made of six pivot joints serially assembled according to a conventional anthropomorphic geometry. The three first active joints form the shoulder and the elbow while the wrist is composed of the three last passive joints. The wrist axes coincide at Point W (see Figure 3). The kinematics are sketched in Figure 3, where Point W is the wrist center while Point T is the probe tip. Note that the position of Point W with respect to the robot base only depends on the three first joint positions while the position of Point T also depends on the positions of the wrist joint. Kinematic models mapping joint positions into Point W or Point T positions follows directly from the Denavit and Hartenberg parameters given in Table.1, (Denavit and Hartenberg, 1955).

• The FREE mode, characterized by high transparency and gravity compensation. This allows for manually positioning the probe under US guidance.
• The LOCKED mode, during which the clinician has his/her hands free to perform the needle placement and the biopsy. Here, it is desired that the robot maintains precisely the target position, while preserving the patient’s safety.

**TABLE 1:** DH parameters of the Apollo robot.

i	*α*_*i*_	*a*_*i*_	*d*_*i*+1_	*θ*_*i*+1_
0	0	0	*θ*_1_	0
1	*π*/2	0	*θ*_2_	0
2	0	25 cm	*θ*_3_	0
3	*π*/2	0	*θ*_4_	30 cm
4	− *π*/4	0	*θ*_5_	0
5	− *π*/2	0	*θ*_6_	0

Apollo thus offers 2 different control modes.

**FIGURE 4:**
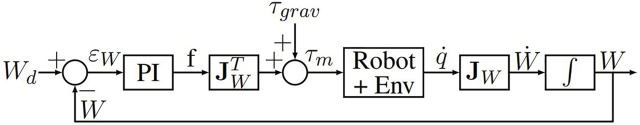
Control law of the robot’s wrist W.

A third mode is aimed at automatically displacing the probe toward a desired anatomical location named “ADJUSTMENT”. This control mode is designed for controlling the desired position of the robot’s wrist center, *W*_*d*_, while also preserving the patient’s safety (see Figure 4).

The control law used is an impedance controller generating forces in response to position errors. Due to the passive wrist, the force transmission model at the *W* point is written:

$$\tau =J_{v1,W}^{\text{T}}\;f\;\;.$$ (1)

Where:

• $\tau ={\left[\tau_{1}\;\tau_{2}\;\tau_{3}\right]}^{\text{T}}$ is the vector of the first 3 torques of each of the 3 motors;
• **f** is the equivalent force to the *W* point;
• **J**_*v*1,*W*_ is the Jacobian matrix associating the velocity of the first three joints of the robot with the cartesian velocity of the robot at the *W* point. By rating abuse, it will now be noted **J**_*W*_.

The control law described in Figure 4 is then written:

$$\tau_{motors}=\tau_{grav}+J_{W}^{\text{T}}\;\left(k_{p}\varepsilon_{W}+k_{i}\int_{0}^{t}\varepsilon_{W}du\right)$$ (2)

With:

• *τ*_*grav*_ corresponds to the torque required to achieve the gravity compensation (Poquet et al., 2013);
• *ɛ*_*W*_ = *W*_*d*_ − *W* is the error between the desired position and the current position of the robot’s wrist *W*;
• *k*_*p*_ and *k*_*i*_ are the proportional gain and the integral gain, respectively, both of which are scalars.

Note that choosing sufficiently low values for *k*_*p*_ and *k*_*i*_ allows low stiffness at the Point *W* with a null static error (slow error cancellation despite perturbations at the insertion point).

It is antagonistic in the context of robot control: usually precision is achieved thanks to high stiffness while in order to respect the safety of the patient, control law requires a low impedance.

### 2.2 Precise Positioning

#### 2.2.1 Problem Description

**FIGURE 5:**
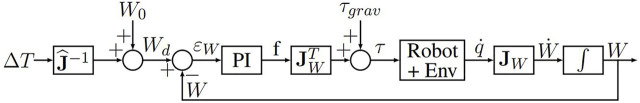
Controller with a well-known mapping between *T* and *W* displacements.

As explained in *Robotic Specification*, the goal of the robot is to improve the surgeon’s precision during the pointing task. The more the pointing task will be accurate the more the biopsy will be relevant. Based on the controller described previously (*Robot Apollo*), when a desired location *T*_*d*_ is specified for the tip, one has to compute the corresponding desired position of the wrist center, *W*_*d*_, which is easily controllable from the three first actuated joints of the robot. As both *T* and *W* belong to the probe (rigid body), one can write :

$$v_{T}=Jv_{W}$$ (3)

where *v*_*T*_ and *v*_*W*_ are the velocities of the probe with respect to the robot base expressed at point *T* and *W* respectively ; **J** is an interaction matrix. The displacement Δ_*T*_ = *T*_*d*_ − *T*_0_, between *T*_*d*_ the desired position of *T* and *T*_0_ the initial position of *T*, is reached if the robot controls the position of *W* according to:

$$W_{d}=W_{0}+\Delta_{W}=W_{0}+J^{-1}\Delta_{T}$$ (4)

where *W*_0_ is the initial position of *W*. The resulting controller is shown Figure 5.

Because of the free wrist, it is not possible to control the position of Point T only based on the robot kinematic model. In the literature (Low and Phee, 2004; Ortmaier and Hirzinger, 2000; Dong and Morel, 2016), free wrist robots are used with the hypothesis that the insertion point is considered as a fixed rotation center of motion (RCM). However, it appears that the mapping from *W* displacements to *T* displacements depends on how the tissues surrounding the insertion site are deformed. In particular, the vagina is far from being precisely described by a fixed fulcrum model. It results in a complex relationship between the position of the robot wrist center W and the tip position T (see Figure 3) as it has been shown in (Smet et al., 2019). Thus, the matrix **J** has to be continuously updated to take it into account.

Therefore, in order to generalize a control law able to adapt to any medical application and more specially in uterine biopsy, it appears necessary to develop an estimator able to take into account the variability of the rotation point throughout an examination. Thus, a precise targeting task can be performed in gynecology and also in other MIS as laparoscopy, urology, etc.

To solve this new problem, two different estimators are developed.

• The first estimator, named Adaptable Lever Arm Model (ALAM), uses the well known lever arm model but continuously updates the ${\hat{J}}_{xx}$ and ${\hat{J}}_{zz}$ values of the interaction matrix.
• The second method is derived from numerical methods for solving nonlinear problems of type y = F(x). This method, named Broyden’s method, allows to estimate directly a Jacobian matrix of size 3 × 3 linking two distinct variables.

It is now necessary to present and test them first in simulation and then on an experimental set-up.

#### 2.2.2 Adaptable Lever Arm Model

When a desired position *T*_*d*_ is specified to the robot for the tip of the instrument, it is necessary to calculate the corresponding desired position of its wrist center, *W*_*d*_, which is controllable from the first three joints of the robot. However, as explained earlier, the interaction between the tip of the instrument *T* and the wrist center of the robot holding the instrument *W* cannot be considered as a perfect lever arm model with a fixed instrument rotation point. Clearly, the correspondence between the displacements of *W* and those of *T* depends on how the tissue surrounding the insertion site deforms. If we consider small movements (local representation), it is reasonable to assume that the behavior is linear, i.e., it is possible to write:

$$\delta T=\hat{J}\delta W=\left(\begin{array}{ccc} {\hat{J}}_{xx} & 0 & 0\\ 0 & 1 & 0\\ 0 & 0 & {\hat{J}}_{zz} \end{array}\right)\delta W\;\;.$$ (5)

This specific structure of **J** comes from the fact that the inserted instrument is assumed to be rigid. Therefore, the displacements of *W* are assumed to be equal to the displacements of *T* on the $\vec{y}$ penetration axis.

As explained in *New Uterine Biopsies Procedure*, during robot manipulation of the instrument, $\hat{J}$ must be continuously updated because it cannot be considered constant. An instantaneous estimation of **J** can be computed from the instantaneous velocities values of *W* and *T*. Indeed, the temporal differentiation of Eq .5 leads to:

$$v_{T}=\hat{J}v_{W}\;\;.$$ (6)

An instantaneous measurement of the velocities **v**_*W*_ and **v**_*T*_ is thus sufficient to identify the 2 unknown elements of **J** because two equations are available (corresponding to the first and third lines of equation.6). However, exploiting the *n* successive measurements of **v**_*T*_ and **v**_*W*_, assuming that they were recorded in sufficiently close configurations to consider that **J** is constant, allows to estimate $\hat{J}$ thanks to a least square optimization. Denoting ${\hat{J}}_{inst}$ the resulting instantaneous estimation of **J**, it is possible to implement the online estimation of ${\hat{J}}_{k}$ at a given time *k* as:

$${\hat{J}}_{k}=\left(1-\lambda \right){\hat{J}}_{k-1}+\lambda {\hat{J}}_{inst},$$ (7)

where *λ* is a scalar gain verifying 0 < *λ* < 1. In practice, *λ* is set small enough to filter out measurement noise and large enough to ensure a satisfactory adaptation rate.

Although this estimator allows to take tissue deformations around the insertion area of the instrument through the patient into account, it is still subject to the diagonal construction assumption of the interaction matrix. As a reminder, this assumption comes from the definition of the minimally invasive surgery instrument insertion problem as a linear annular connection.

However, making this assumption about the construction of $\hat{J}$ means that the forces on the walls of the insertion zone are decoupled along each axis and do not interfere with each other. It is complicated to confirm this hypothesis from an anatomical point of view because of the difference in elasticity between each insertion zone (uterus, anus, trocar, etc.) of each patient. Therefore, an estimator based on non-linear systems is developed in order to get rid of the assumption of construction of the interaction matrix used until now. It is thus possible to identify any interaction matrix of the form:

$$J=\left(\begin{array}{ccc} J_{xx} & J_{xy} & J_{xz}\\ J_{yx} & J_{yy} & J_{yz}\\ J_{zx} & J_{zy} & J_{zz} \end{array}\right).$$ (8)

#### 2.2.3 Broyden Model

System identification is a branch of automatic control that consists in obtaining a mathematical model of a system from measurements on it. The problem discussed here is written as the resolution of a nonlinear system.

In keyhole surgery, errors in the estimation of the instrument-patient interaction lead to a deterioration of the closed-loop behavior when the *T* point is returned to the controller. This can have a significant impact in real-world situations, where the interaction cannot be modeled as a support point and not known precisely in advance. For example, in (Chalard et al., 2018), it was shown that the insertion point can be moved more than 20 mm during a prostate biopsy. In (Smet et al., 2019) it is shown that manipulation of the uterus with an instrument through the vagina during surgery cannot be modeled as a pivot joint.

In fact, the mapping of **J** displacements from *W* to *T* depends on how the tissues surrounding the insertion site deform. As the deformation of the tissues cannot be modeled and depends on the insertion zone (uterus, anus, stomach, etc.), the interaction between $\dot{W}$ and $\dot{T}$ can be modeled with the general shape:

$$J=\left(\begin{array}{ccc} J_{xx} & J_{xy} & J_{xz}\\ J_{yx} & J_{yy} & J_{yz}\\ J_{zx} & J_{zy} & J_{zz} \end{array}\right).$$ (9)

Moreover, to take the deformation of the tissues when the instrument is handled into account, **J** must be continuously estimated as $\hat{J}$ . In the previous section (*Adaptable Lever Arm Model*), the problem was partially solved by assuming that the structure of **J** can be simplified.

The problem stated above is a nonlinear optimization problem since the matrix **J** depends on the position *W* and the unknown environment. In the literature, the most common numerical method to solve this kind of problem is the Newton method. More particularly, when it is necessary to estimate a Jacobian matrix, the Broyden method is used. This is an iterative method that can be used to estimate the Jacobian matrix (Mansard et al., 2006) of a robot. This method uses an initial guess to generate an improvement sequence of approximate solutions. It gives good results assuming that the initial value is not too far from the actual value. In addition, this method has a low computational cost that allows for online estimation.

Based on the Broyden method (Broyden, 1965), it is possible to use the Broyden matrix by applying it directly to the context of medical robotics. Thus, the estimated matrix $\hat{J_{k}}$ is computed such that:

$$\hat{J_{k}}={\hat{J}}_{k-1}+\alpha \frac{\delta T_{k}-{\hat{J}}_{k-1}.\delta W_{k}}{\|\delta W_{k}{\|}_{2}^{2}}.\delta W_{k}^{T}$$ (10)

where:

• *δT*_*k*_ = *T*_*k*_ − *T*_*k*−1_ is the measured displacement of the instrument tip *T* between the two iterations.
• *δW*_*k*_ = *W*_*k*_ − *W*_*k*−1_ is the measured displacement of the robot end effector *W* between the two iterations.
• *α* is a scalar gain.

The parameter *α* is a scalar gain between 0 and 1 which defines the update speed of the Algorithm. When setting this parameter, a compromise must be found between convergence speed and robustness. If the variation of the input data is too small or null, the computation can become unstable. To avoid this instability, it is necessary to verify:

$$\delta W_{k}^{T}.\delta W_{k}=\|\delta W_{k}{\|}_{2}^{2}\neq 0$$ (11)

A threshold is then introduced to ensure that the previous condition is verified:

$$\|\delta W_{k}{\|}_{2}\geq r_{\varepsilon }$$ (12)

where *r*_*ɛ*_ must be set according to the application. If the threshold is not reached, the matrix is not updated and:

$${\hat{J}}_{k}={\hat{J}}_{k-1}$$ (13)

## 3 Simulation Results

### 3.1 Identification Process

To choose the model that will give an identification as close as possible to the real interaction matrix, there is a multi-step identification procedure (Rachad et al., 2015), (Liu et al., 2015) that allows to test and compare different identification model structures. All categories are defined as:

• Test protocol: it needs sufficient data that represent the dynamics of the system. Pseudo-random signals are typically used as input to the system in order to have a good excitation of the system;
• Measurement and signal processing;
• Choice of model structure: choice of model type, initial conditions and convergence factor;
• Parametric identification: use of a parametric optimization algorithm;
• Validation of the model: execution of verification tests, analysis of the results;

To find the better identification model, the procedure consists in :

• first step: performing measurement and signal processing based on a test protocol;
• second step: extracting a parametric identification based on the measures in step one and the choice of a model;
• third step: validating the model.
• updating step : if the model is not validated, it is possible to repeat the procedure by updating the choice of the model, the test protocol and the parametric identification until you find the correct identification.

Based on this procedure, an experimental protocol is developed.

### 3.2 Data Acquisition

**FIGURE 6:**
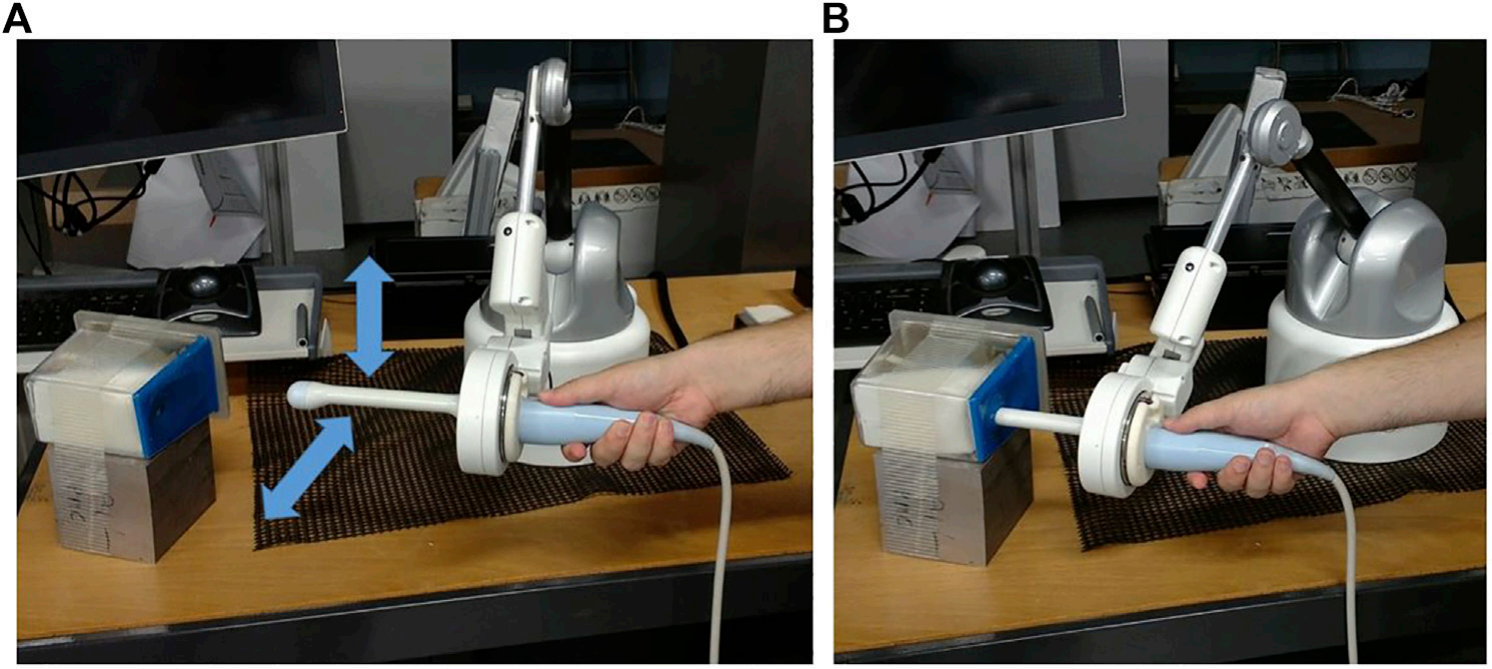
Experimental setup without environmental constraints **(A)**, in an anatomical phantom **(B)**.

Two experiments were conducted to validate the proposed estimation method. During these experiments, the instrument is moved in comanipulation with the Apollo robot (the robot being in free mode). The positions of *T* and *W* are measured thanks to the sensors of the robot and acquired. Two experiments have been performed:

• For the first experiment, no environment applies any constraint to the instrument. The user freely manipulates the probe according to perpendicular translations while maintaining a constant orientation as illustrated in Figure 6). In this case, the displacement of *T* is equal to the displacement of *W*. This experiment will hereafter be called “movement 1”.
• In the second experiment, the instrument is inserted into an anatomical phantom. The user manipulates the instrument as they would during a gynecological or prostate examination (Figure 6). This experiment will be called ‘”movement 2”.

After the experiment, the data are exploited in post-processing. In order to verify that the two estimators are performing well, they are both tested on the two experiments performed.

To validate their behavior, they are implemented in a simulation using the software Matlab. By recovering the positions of the robot end effector (Point W) and the positions of the instrument tip (Point T) it is possible to reconstruct a position of the instrument tip, noted *T*_rec_. It is calculated from the measured position of *W* and the matrix estimated by each of the two estimators, see Algorithm 1 and Algorithm 2.

**Algorithm 1:** Reconstruction of the instrument tip based on the Adaptable Lever Arm Model (10 ms).

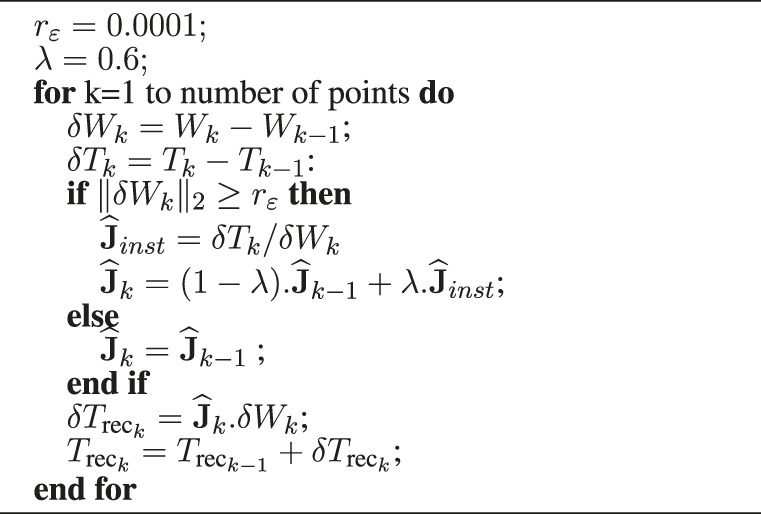

**Algorithm 2:** Reconstruction of the instrument tip based on the Broyden algorithm (10 ms).

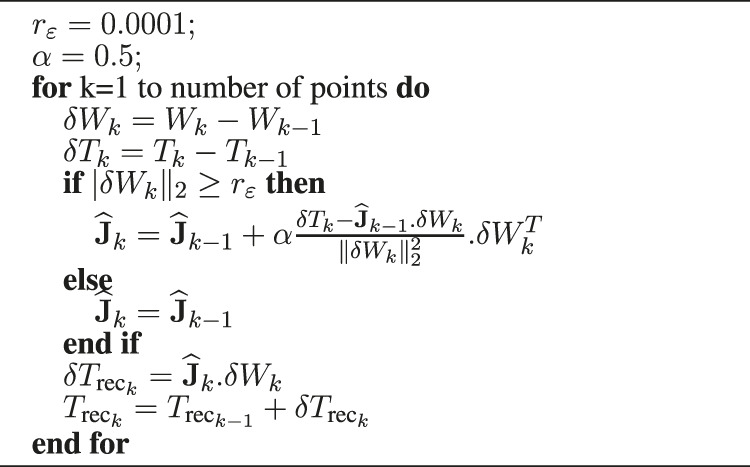

Whatever the experiment and the estimator, the interaction matrix $\hat{J}$ is initialized as a fixed RCM model. With a manual calibration, ${\hat{J}}_{init}$ is defined as.

$${\hat{J}}_{init}=\left(\begin{array}{ccc} -0.15 & 0 & 0\\ 0 & 1 & 0\\ 0 & 0 & -0.15 \end{array}\right).$$ (14)

Also, note that the values *r*_*ɛ*_, *α* and *λ* are empirically chosen as:

• *r*_*ɛ*_ = 0.0001 (m);
• *λ* = 0.6;
• *α* = 0.5;

In particular, the *r*_*ɛ*_ value was set based on physical limits. Indeed, as defined in the previous section (*Broyden Model*), this threshold affects the Jacobian update based on the input data. In our case, the input data is the displacement of the point W. It was decided to update the Jacobian matrix if the robot is moving. Therefore, to take this into account, the *r*_*ɛ*_ value was set to one tenth of a millimeter between each 10 milliseconds. Then, thanks to an iterative method, the *α* and *λ* values were fixed in order to find a compromise between the convergence speed and the error reconstruction. Indeed, the closer their values are to 1, the higher the convergence speed of the algorithm. However, it strongly impacted by the input variation and conversely if the values of *λ* and *α* are close to 0.

The reconstructed positions of the instrument tip *T*_rec_ are then compared to the actual position of the instrument tip measured by the robot, denoted $T_{{meas}_{k}}$ . The reconstruction error $\varepsilon_{T_{k}}$ is defined as:

$$\varepsilon_{T_{k}}=T_{{meas}_{k}}-T_{{\text{rec}}_{k}}$$ (15)

In this way, the smaller the $\varepsilon_{T_{k}}$ error is, the more the algorithm is able to artificially reconstruct the position of the instrument tip. In practice, this means that if $\varepsilon_{T_{k}}$ tends to zero at any time, the estimators are able to identify the interaction between the part of the instrument located inside the patient (point T) and the one located outside (point W). Thus the estimators tend to the value of the real interaction matrix **J**.

### 3.3 Reconstruction of the Interaction Matrix Based on ALAM and Broyden Method

**FIGURE 7:**
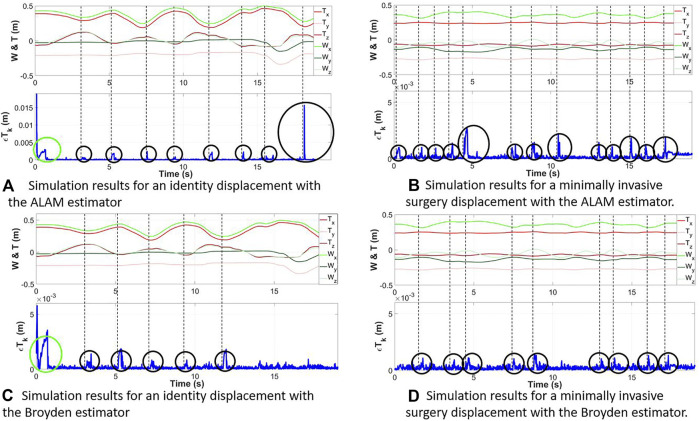
Measured position of the robot wrist (green) and the instrument tip (red) during a displacement. Evolution of the reconstruction error $\varepsilon_{T_{k}}$ (blue) during the two experiments with the ALAM **(A,B)** and Broyden **(C,D)** estimators.

The results of the four experiments are shown in the Figure 7.

For the movement 1:

• the average reconstructed error ( $\varepsilon_{T_{k}}$ ) with the Adaptive Lever Arm Model is less than 0.721 mm (standard deviation 1.09 mm).
• the mean reconstructed error ( $\varepsilon_{T_{k}}$ ) with the Broyden model is less than 0.257 mm (standard deviation 0.477 mm).

Moreover, for the movement 2:

• the average error ( $\varepsilon_{T_{k}}$ ) with the Adaptive Lever Arm Model is less than 0.232 mm (standard deviation 0.314 mm).
• the mean error ( $\varepsilon_{T_{k}}$ ) with the Broyden model is less than 0.235 mm (standard deviation 0.191 mm).

Whatever the movement or the chosen estimator, we can note the presence of error peaks (black circle) in Figure 7. They appear in the case of a sudden change in the direction of motion, highlighted by the dotted black vertical line. However, Figure 7 show that the two proposed methods succeed in cancelling the error after a few iterations.

Moreover, taking into account the particular link of the W and T points for the movement 1 (the wrist of the robot and the tip of the probe have the same speed), the green circle visible on Figures 7A,C highlights a peak in the reconstruction error due to the initial value of $\hat{J}$ which is totally different from the real value.

Concerning the ‘movement 2′, the changes of direction are smoother because of the constraint of the insertion which has the effect of making the peaks of reconstruction error almost disappear. This can also be seen from the mean values of the reconstruction errors as well as their standard deviation for ‘movement 2′ which are, for both estimators, lower than for ‘movement 1’.

### 3.4 Discussion

**FIGURE 8:**
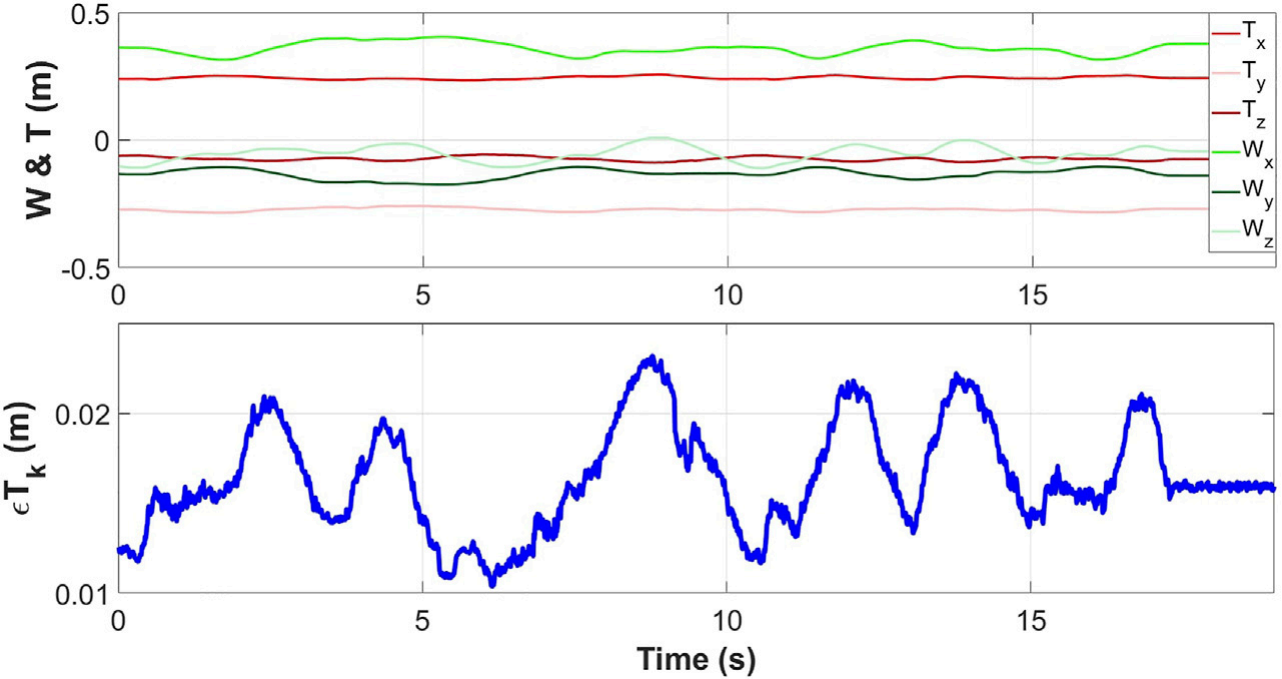
Measured position of the robot wrist (green) and the instrument tip (red) during a displacement. Offline reconstructed error $\varepsilon_{T_{k}}$ (blue) during the ‘motion 2′ determined with the fixed lever arm model.

As a comparison, a simulation using a fixed lever arm model was tested. This model is one of the most used in the literature and was implemented based on a pre-test placement (Wei et al., 2005). It was only implemented on the “movement 2” which corresponds to a mini-invasive type of movement. The results of this simulation can be found in Figure 8. The average error ( $\varepsilon_{T_{k}}$ ) with the fixed Lever Arm Model is 16.4 mm (standard deviation 2.9 mm).

Regardless of the movement, based on the mean error (and standard deviation) and overall performance (Figure 7), it can be concluded that both of the proposed methods for estimating the interaction matrix continuously are more effective than the fixed lever arm model. They both correctly estimated the position of the instrument tip at any time. To face these problems of non-fixed or even non-existent insertion point, it is proposed to implement the estimators tested in simulation on an experimental set-up to validate their interest.

## 4 Results

### 4.1 Experimental Set-Up

**FIGURE 9:**
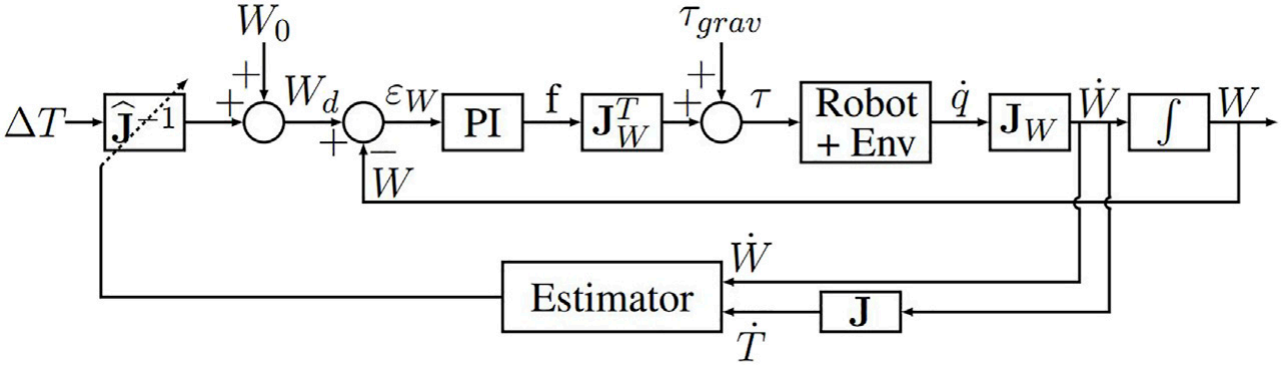
Open loop control of the Apollo robot for automatic fine movements of the instrument tip taking into account the elasticity of the environment.

**FIGURE 10:**
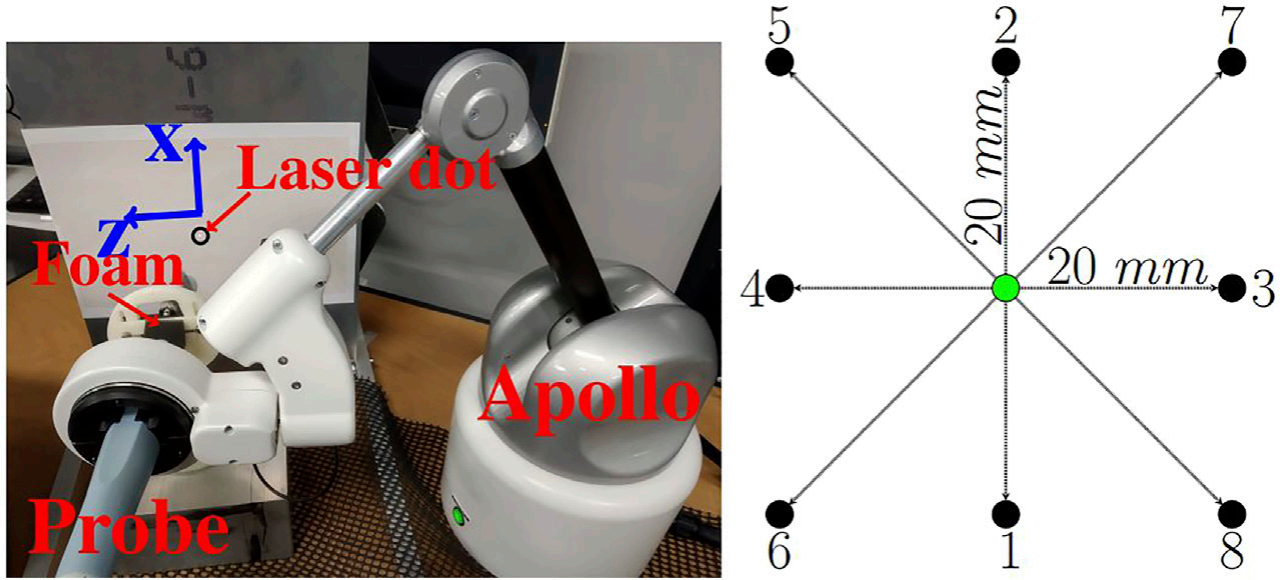
Set-up and targets.

As explained in *Problem Description*, it is possible to realize fine automatic movements by controlling the wrist of the robot thanks to the controller detailed previously (Figure 6). Moreover, as highlighted in *Precise Positioning*, to reach a target with the probe tip (point T) by controlling the robot wrist (point W), it is necessary to accurately estimate the **J** interaction between W and point T (see Figure 9). The estimators are thus implemented in the open-loop control described in *Problem Description*. Indeed, by implementing the two estimators in the open loop control and by comparing them to a classical calibration estimate at the beginning of the test, it is enough to look at the final positioning error to know the most efficient and reliable method. Based on this consideration, the accuracy of the estimator could be measured through real displacements of the probe. Indeed, by controlling the wrist of the robot, the better the estimation of the interaction matrix, the closer the final position T will be to the desired position T. To quantify the displacements, a laser is attached to the probe. The laser is pointed on a graph paper and the pointing error is thus recorded at the end of the probe movement (see Figure 10).

In order to ensure the accuracy of the estimators, the targets are chosen to be able to verify the correlation and/or the decorrelation of the displacements due to tissue deformations. Indeed, it is interesting to perform displacements along a single axis and also along 2 axes simultaneously. The targets are defined as the corners of the square (Targets 5, 6, 7 and 8, see Figure 10). The square is designed as a 40 mm side square. Each middle of the sides of the square forms the other 4 targets (Targets 1, 2, 3 and 4, see Figure 10). For each targeting task, the starting point is the center of the square. This allows movements of different lengths (20 and 28.3 mm). In addition, the square is covered three times, so each target was reached three times. Moreover, in order to simulate anatomical constraints (elasticity, no RCM, etc), the insertion point is covered with foam (see Figure 10). Thus, regardless of the displacement, the interaction between the W and T points is still unknown. Note that in order to be relevant with the Sec.2.2.1, the initial value of the interaction matrix **J** is always the same.

In order to be able to position our work with respect to the existing literature on the positioning of surgical instruments during prostate or uterine surgery, a fixed RCM type control found in the literature (Yip et al., 2017) was also implemented (see Figure 5). In this configuration, the initialization of the interaction matrix $\hat{J}$ is fixed a :

$${\hat{J}}_{\mathrm{fi}xe-br}=\left(\begin{array}{ccc} -1.55 & 0 & 0\\ 0 & 1 & 0\\ 0 & 0 & -1.55 \end{array}\right).$$ (16)

It should be noted that for the displacements with the Adaptive Lever Arm Model (ALAM) the displacements will be restricted to targets 1, 2, 3 and 4 with displacements of 10 mm. Moreover, as the experiments were not performed at the same time as those for the Broyden model, the initial value of the initialization matrix differs and is fixed at:

$${\hat{J}}_{\mathrm{fi}xe-lam}=\left(\begin{array}{ccc} -0.8 & 0 & 0\\ 0 & 1 & 0\\ 0 & 0 & -0.8 \end{array}\right).$$ (17)

In order to standardize the results obtained for each of the two estimators, they are both compared to a classical calibration method performed with robotic systems having RCM (as it has been defined in *Discussion*).

### 4.2 Evaluation of the Two Estimators

#### 4.2.1 Adaptable Lever Arm Model

Table.2 contains the different errors obtained by using a constant $\hat{J}$ interaction matrix and those obtained with the ALAM estimator which allows to update $\hat{J}$ . The error calculated for each of the 8 steps is the absolute value of the difference between 10 mm (desired displacement) and the actual displacement measured at point *T*. The average error when using a constant $\hat{J}$ is 2.36 mm while it is reduced to 0.81 mm with a continuously updated $\hat{J}$ .

**TABLE 2:** Displacement standard for each transverse side given by Apollo with $\hat{J}$ constant and updated.

Δ*T* desired of 10 mm	with $\hat{J}$ constant	with $\hat{J}$ updated
Displacement of T	7.5	12.2
	6.4	10.5
along $\vec{x}$	8.5	10.8
(mm)	8.1	10.3
Displacement of T	5.7	11.1
	6.1	10.2
along $\vec{z}$	9.8	9.8
(mm)	9	8.8
Average error	**2.36(23.6*%*)**	**0.81(8.1*%*)**

Clearly, consideration of an anisotropic pattern and online pattern identification significantly reduces the targeting error of the instrument tip.

#### 4.2.2 Broyden Model

**FIGURE 11:**
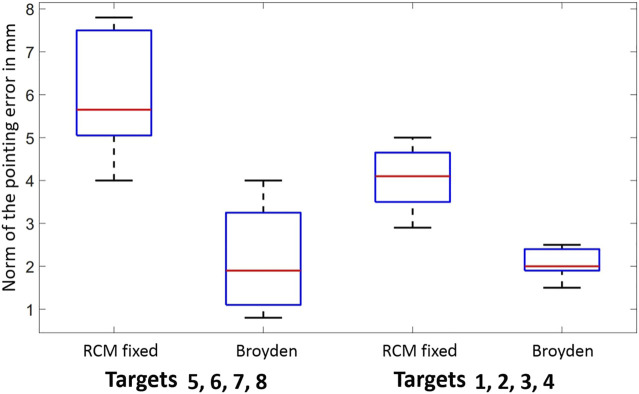
Average error of the scoring task with the RCM Fixed model and the Broyden Model.

Figure 11 compares the errors obtained using a fixed RCM model and a continuously updated $\hat{J}$ interaction matrix. The error calculated for each of the 24 steps is the absolute value of the difference between the desired displacement and the actual displacement measured at point T. In Figure 11, the displacements correspond to two classes. The first class includes the 12 displacements of 20 mm along the $\vec{x}$ and $\vec{z}$ axes of the probe (targets 1, 2, 3 and 4). The second class includes the other 12 displacements that move simultaneously along the $\vec{x}$ and $\vec{z}$ axes of the probe (targets 5, 6, 7 and 8).

It appears that the controller based on the continuous estimation of the interaction matrix using the Broyden method is better than the controller based on the fixed RCM.

Specifically, for targets 1, 2, 3, and 4 the Broyden controller reduces the pointing error by more than 2 mm for a 20 mm displacement compared to the fixed RCM controller. In addition, for targets 5, 6, 7, and 8, we reduce the pointing error by more than 3 mm. Overall, for all displacements, the accuracy of the pointing task is improved by 11.8*%* with the Broyden controller. In detail, the accuracy for all moves is:

• Broyden controller accuracy = 91.1*%*
• Fixed RCM controller accuracy = 79.3*%*

### 4.3 Discussion

Clearly, taking a numerical model based on the Broyden method to continuously identify the **J** interatcion matrix into account significantly reduces the pointing error. Although both estimators improve the open-loop control with our set-up in a similar way, the choice of the Broyden model is selected. Indeed, although trying to reproduce the behavior of the vagina anatomy as well as possible, the set-up seems to be more similar to the conditions of prostate biopsy or laparoscopy. In this context both estimators improve the overall behavior of the open-loop control compared to the fixed lever arm model. However, if we look at the description of the uterus, the Broyden algorithm is more recommended than the adaptive lever arm model. Indeed, in (Smet et al., 2019), it is shown that it is impossible to consider the insertion zone as a rotation point. Therefore, the estimation model based on an adaptive lever arm model appears less relevant for this medical examination.

## 5 Discussion

Therefore, after testing both estimators in simulation and in experimental set-ups, the Broyden method clearly appears as the better solution. It cans take the deformation of the insertion area into account during MIS in order to precisely position the instrument tip.

**FIGURE 12:**
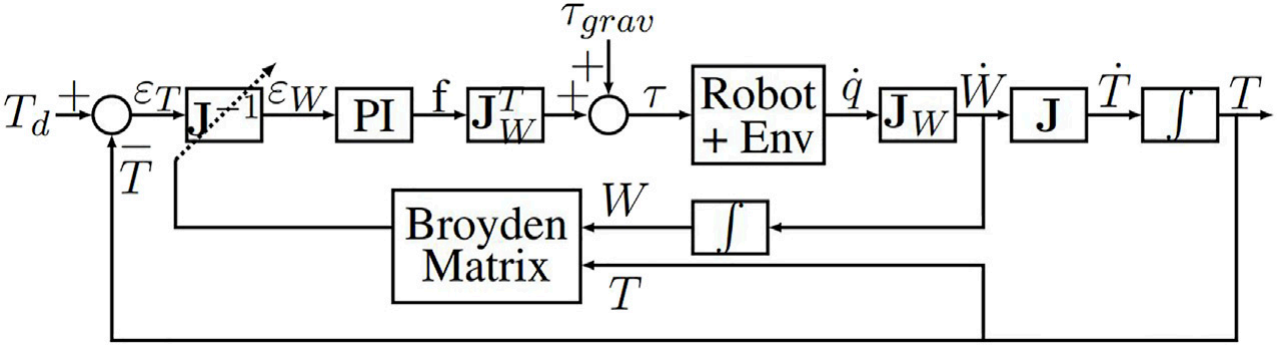
Closed loop control of the Apollo robot for automatic fine movements of the instrument tip taking the elasticity of the environment into account.

Moreover, the best solution to reach a target with a robot is to use a close loop controller on the instrument tip. It guarantees a zero error for the targeting tasks of the instrument tip. Closing the loop at point *T* is then possible with the following control law (see Figure 12):

$$\tau =\tau_{grav}+J_{W}^{\text{T}}\;\left(k_{p}{\hat{J}}^{-1}\varepsilon_{T}+k_{i}\int_{0}^{t}{\hat{J}}^{-1}\varepsilon_{T}du\right)$$ (18)

**FIGURE 13:**
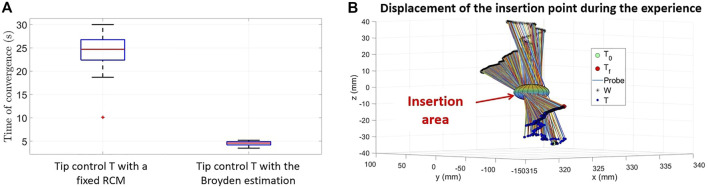
**(A)** Convergence time for the 40 trials (Controller without adaptation vs Controller with adaptation) and **(B)** Highlighting of an “insertion area” in the developed experimental set-up.

With such a controller, due to the integration of *ɛ*_*T*_, a null error at point *T* is guaranteed providing that the system remains stable. The control law and the results are described in (Chalard et al., 2020). This leads to the conclusion that controlling fine automatic displacements of the instrument tip in close loop control by taking the elasticity/deformations of the insertion zone into account thanks to the Broyden estimator allows to increase tenfold the performances of the targeting task. Indeed, with a continuous estimation of the **J** interaction matrix, the convergence towards the desired target is achieved on average 5 times faster than with an estimated interaction matrix considered fixed (see Figure 13).

It should also be noted that, as with the movements made with the open loop controllers, the insertion point around which the instrument rotates instantaneously is not fixed. As shown in Figure 13 it can indeed move in a 2 cm wide area.

## Conclusion

This paper focuses on the definition of the interaction matrix **J** and its importance for the realization of a precise control in minimally invasive robotic surgery and more particularly for uterus biopsy. Indeed, it is shown that if this matrix is badly estimated, it cans have undesirable consequences on the control of the robot and sometimes lead to a divergence of the system. In the literature, the most common method to identify **J** is to consider the interaction between the instrument held by the robot and the patient’s body as an annular linear link. This kinematic constraint restricted the working space to 4 degrees of freedom. Considering the insertion point as fixed, many robots have been developed with an offset center of rotation (RCM). These can either be mechanically imposed and are then called “active” or directly imposed by the anatomy of the insertion point and considered as ‘passive’. In the first case, the surgeon must manually match the remote rotation point of the robot with the rotation point of the instrument imposed by the anatomy. In the second case, the rotation point of the instrument is unknown to the robot. To overcome this problem, 6 degrees of freedom robots have been designed. Thanks to their sensor data, they are able to reconstruct a mean rotation point close to the actual rotation point of the instrument using a least squares algorithm (Dong and Morel, 2016). Although each of these options has its advantages and disadvantages, the biggest issue lies in the assumption of their design. Indeed, studies have shown that the actual point of rotation of the instrument cannot be considered as fixed throughout a surgery. It is subject to variations in position due, among other things, to the elasticity of the tissues surrounding the insertion zone of the instrument.

The models of the literature are till sufficient to perform tasks requiring little precision (coarse displacement of an endoscope controlled by the surgeon) and more generally to perform tasks involving direct control of the robot by the surgeon. However they cannot be applied to a task such as fine automatic displacement where the surgeon no longer intervenes in the control loop.

To account for this new assumption, two models (Adaptive Lever Arm Model and Broyden) have been developed. They can continuously identify the interaction matrix linking the instrument tip velocity with the robot effector velocity. In this paper, simulations on post-processed robot’s data have:

• showed the importance of taking into account the displacement of the rotation point of the instrument during a manipulation;
• validated the working principle of the proposed estimators to continuously identify the **J** interaction matrix.

In order to experimentally validate the results obtained in simulation, these two estimation models were then implemented on the Apollo robot to validate them on an experimental set-up.

Both estimators were implemented in an open loop control of the probe tip in order to evaluate their performance against the RCM solution found in the literature. From an experimental set-up simulating the insertion of an endocavity probe through an unknown sinking, automatic fine displacements are then re-assembled. Although both estimators are better than the one developed in the literature, only the estimator based on Broyden’s method is retained. Indeed, the anatomical constraints related to the biopsy of the uterus do not allow to define the displacement of the probe from a rotation of the probe around a variable point. Therefore, although its results are similar to Broyden’s model, the Adaptable Lever Arm Model (ALAM) construction hypothesis appears inconsistent with our application.

Although experimentally validated on an *in vitro* laboratory set-up and implemented on a close loop controller, the Broyden method will be necessary from now on to carry out an experimental set-up reproducing the vagina and the uterus as faithfully as possible. It needs to be tested and validated on an ultra-realistic set-up in order to hope to carry out *in-vivo* tests.

## Data Availability

The original contributions presented in the study are included in the article/supplementary material, further inquiries can be directed to the corresponding author.
